# The Long-Term Results of Frontalis Suspension Using Autogenous Fascia Lata in Children with Congenital Ptosis under 3 Years Old

**DOI:** 10.1155/2010/609462

**Published:** 2009-12-24

**Authors:** Lale Kozer Bilgin, Baris Yeniad

**Affiliations:** Department of Ophthalmology, Istanbul Faculty of Medicine, Istanbul University, Capa-Sehremini, 34390 Istanbul, Turkey

## Abstract

*Purpose*. To describe the long-term results of frontalis suspension using autogenous fascia lata in children with congenital ptosis under 3 years old. *Methods*. Forty three-eyes of 35 patients were enrolled in the study. Frontalis suspension using autogenous fascia lata was performed in all patients. The postoperative eyelid level, ptosis recurrence, visual acuity, and cosmetic results were evaluated. *Results*. The mean age of the patients was 16.8 ± 9 months (7–36 months). The mean follow-up time was 52.8 ± 15 months (14–95 months). All patients had good (ptosis <2 mm) or moderate (2-3 mm ptosis) eyelid level after the operation. All patients achieved satisfactory cosmetic results. Succesfull harvesting was performed in all cases and no additional materials or surgical manipulation were needed during the surgery. *Conclusion*. Frontalis suspension using autogenous fascia lata can be used in children under 3 years old without harvesting problems. Surgical experience and good knowledge of anatomy are important factors for successful results after the surgery.

## 1. Introduction

Congenital ptosis has negative effects on the psychological development of a child [1, 2]. Abnormal head posturing develops in bilateral cases [3, 4], and it can cause deprivation amblyopia, especially in unilateral cases [3, 4]. Therefore, congenital ptosis should be corrected in the early years of childhood, and amblyopia treatment should begin as soon as the diagnosis is established. Although its etiology is unknown, myogenic ptosis is the most common form of congenital ptosis [3]. In myogenic ptosis, either there is no levator function or the levator function is less than 4 mm [3]. In these patients, the diagnosis is established if the upper lid cannot come down to lower limbus and stays in its upper position when looking down [4]. If there is no levator function or the levator function is less than 4 mm, the most effective surgical approach is suspension of the upper lid to the frontal muscle [3]; in this way, the upper eyelid is elevated upon raising the brow.

Currently, autogenous fascia lata is considered to be the best suspension material [2, 3]. Using fresh autogenous fascia lata as a frontal suspension method was first described by Payr [1] in 1909 and performed by Wright [2] in 1922. Crawford [3] modified the technique in 1956. Since using autogenous fascia lata requires a second surgical procedure and experience, in addition to extending the procedure, suture materials such as banked fascia lata [4], palmaris longus tendon [5], polytetrafluoroethylene [6], silicone band [7], 4-0 nylon [8], mersilene mesh [9], and dura [9] are all used. Still, all surgeons agree that the most successful material is autogenous fascia lata, and this technique has the lowest complication rate [9–11]. 

Fascia lata mostly completes its development in the first year of the life [12]. When the length of the upper leg is approximately 20 cm, the length of the fascia lata is sufficient for frontal suspension material. In this study, the frontal suspension technique with autogenous fascia lata was performed as surgical procedure on patients younger than 3 years old. The cases were studied according to long-term visual and surgical results. 

## 2. Materials and Methods

In this study frontal suspension with autogenous fascia lata was performed by the Crawford method on 43 eyes of 35 patients who were 7 to 36 months old (mean age 25 ± 9 months, 18 girls and 17 boys). 

Full ophthalmic and orthoptic examinations were performed before the surgical procedure in all cases. All patients were operated on due to visual axis obstruction or abnormal head posture. The position of the upper eyelid margin (as mm) was measured while the patient looked in primary gaze, and abnormal head position was estimated. Levator muscle function was estimated by measuring the excursion of the eyelid margin as the patient was following shiny objects from a downward gaze to an upward gaze, while inhibiting the action of the frontalis muscle by applying pressure over the brow. Lagophthalmos assessment and the Bell reflex were checked after a detailed conversation with the parents. 

All refractive errors were corrected after a detailed examination with cyclopentolate 1%. Two-hour occlusion therapy was started in unilateral ptosis cases until the surgery date, while alternating occlusion was performed in the binocular cases. The patients under 3 years old with poor levator function (<4 mm) were included in the study. The exclusion criteria were; levator function >4 mm, patients >3 years old, and patients with no Bell phenomena.

## 3. Surgical Procedure

**Figure 1:**
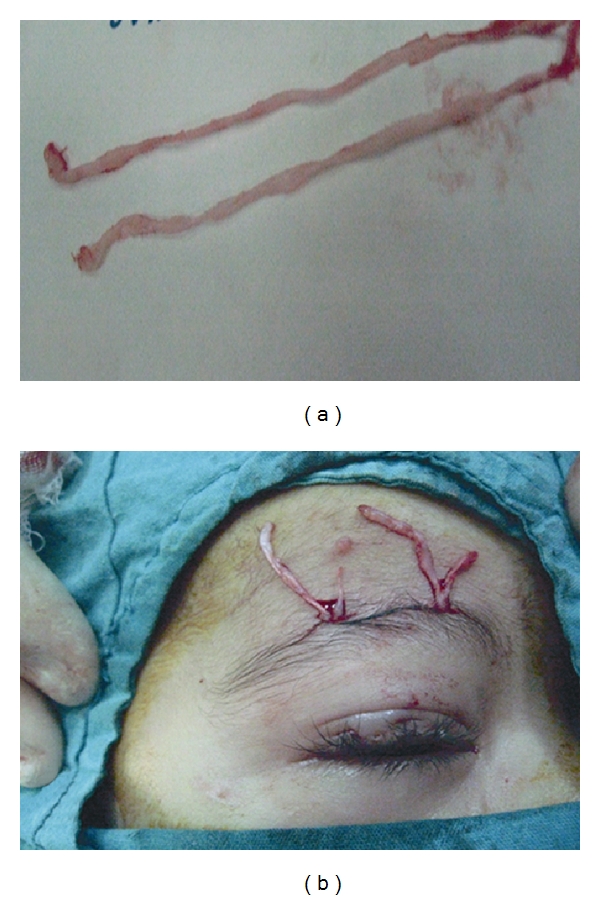
(a) Fascia lata strips after harvesting process, (b) Fascia lata strips were passed through the incision sites.

All surgical procedures were performed under general anesthesia. Harvesting of the fascia lata and frontalis suspension were performed by the same surgeon (LKB). After antiseptic preparation, an 8–10 cm vertical skin incision was made with a blade between the anterior superior iliac crest and the lateral condyle extending downward to the knee. The incision was carried down through the subcutaneous tissue and fat with dissection, and the fascia lata was then revealed. The required portion of the fascia lata (6–8 mm of width) was clutched with forceps (for one eye). The lower end of this portion was cut to release the fascia lata. The desired length was attained by placing stripper all along the incision (approximately 10–12 cm, upwards). The fascia lata was clinched between the skin and the striptor and was cut by the sharp end of the striptor. The subcutaneous tissues were closed with 5/0 Vicryl and skin with 5/0 silk sutures. The fascia lata was placed in physiological serum and cleaned under a microscope. Finally, fascia lata strips of 2-3 mm thickness, 1-2 mm in width, and 8–10 cm in length were prepared (Figure 1(a)). 

The frontalis suspension procedure was performed by the Crawford technique [3]. Three stab incisions of 2-3 mm above the upper eyelid margin were performed. Two stab incisions above the eyebrow and one centrally in the forehead were also made. Deep skin incisions were made in all regions. The prepared fascia lata strips were transferred to the incision sites at the upper lid and eyebrow incisions, under the orbicularis oculi muscle, over the tarsus and orbital septum using a Wright needle (Figure 1(b)). After adjusting the height of the eyelid, the ends were knotted and sutured with 6/0 Vicryl. The height of the eyelid was adjusted 1 mm over the superior limbus for bilateral cases; in unilateral cases, however, it was adjusted to be symmetrical to the other eyelid. The incision sites were closed with 6/0 Vicryl sutures. Frost sutures were then placed in the lower eyelid, and the surgical procedure was finished. 

Topical antibiotics and lubricating eye drops were given to the patients after the operations. The patients were monitored for 1 week after the operation and evaluated every month for the first 6 months. Thereafter, the patients were evaluated every 3 months. 

## 4. Results

**Figure 2:**
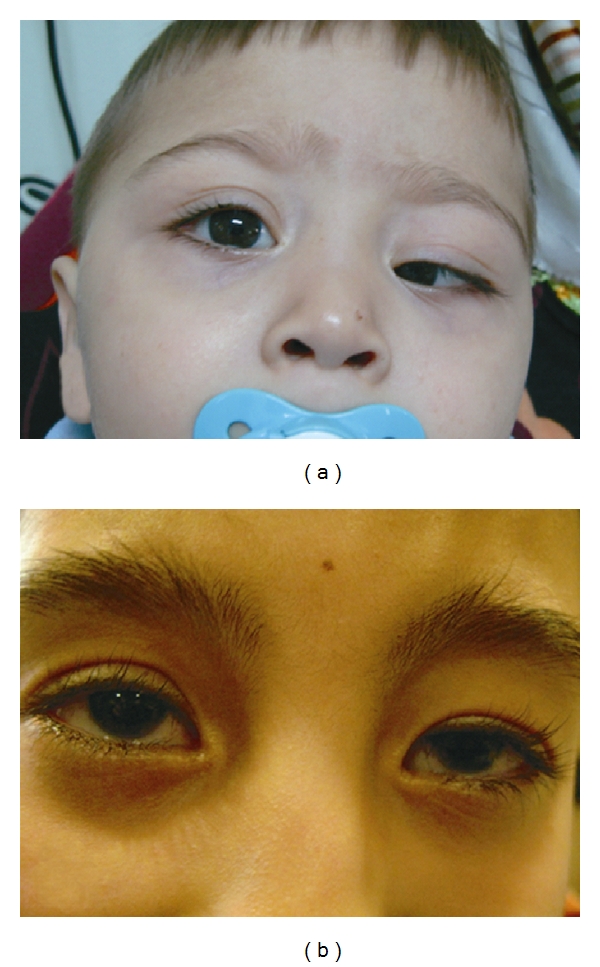
(a), The preoperative picture of the patient with congenital ptosis of the left eye, (b), Six years after the surgery; the patient shows good lid level position.

**Figure 3:**
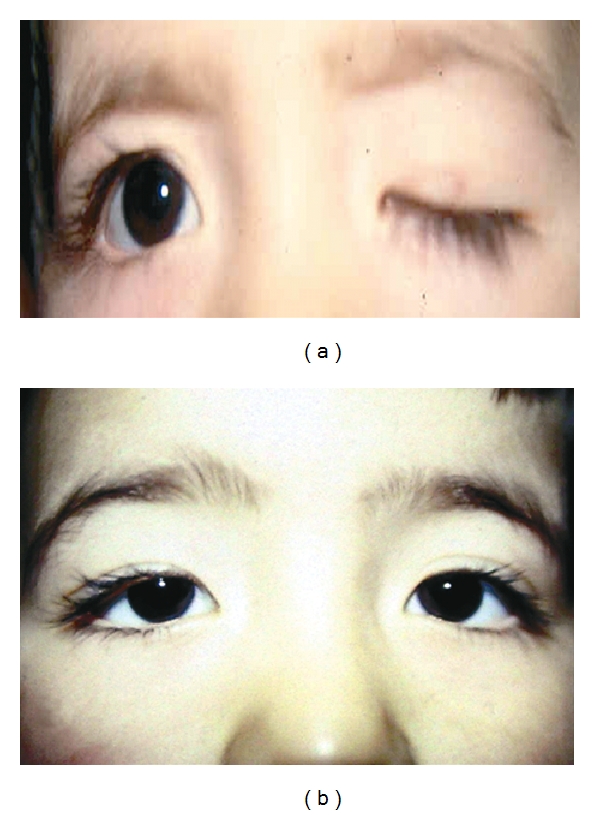
(a) The severe ptosis of the left eye preoperatively, (b) Good eyelid level position three years after the surgery.

**Figure 4:**
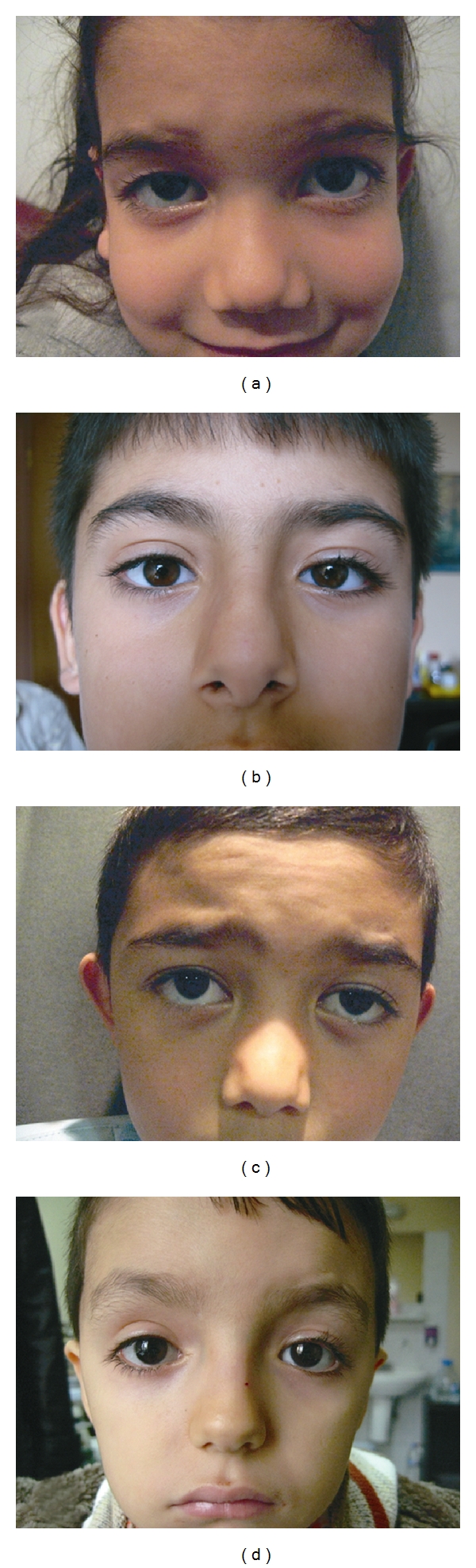
((a)–(d)) Postoperative pictures of the several patients who underwent frontalis suspension using autogenous fascia lata.

The mean age of the cases was 16.8 ± 9 months (7–36 months), and the mean follow-up period was 52.8 ± 15 months (14–95 months). The results were classified as good, moderate, or poor according to the following criteria: a good result was residual ptosis less than 2 mm; a moderate result, 2-3 mm; and a poor result, more than 3 mm. There were 33 eyes with a good result and 9 eyes with a moderate result at the final follow-up (Figures 2, 3 and 4). The second surgical procedure (shortening of the fascia lata) was not performed since the visual axis was not obstructed in any of the cases, and the families were satisfied with the results. Residual ptosis of more than 3 mm was not seen in any of the patients. In one case an overcorrection of 1 mm was examined, but it was considered to be cosmetically acceptable. 

No infection, lagophthalmos, and related corneal problems, or fistula formation at the incision site was seen postoperatively, and no ptosis recurrence was observed. In addition, no neurological deficits or functional loss of the leg was detected. The scars were small and acceptable in all patients. 

Strabismus was detected in 8 of the 35 cases at the follow-up examination, and strabismus treatment was carried out. Occlusion treatment was also continued in all cases after the operation. The visual acuity of the 19 cases (23 eyes) was evaluated after the regular follow-up period of 85 ± 16 months according to age group. The mean visual acuity for cases operated at 1-2 years (10 eyes) was 0.82 ± 0.12, and for cases operated at 2-3 years (13 eyes), it was 0.71 ± 0.16 according to Senellen acuity chart. At the last examination, visual acuity was 0.5 or greater according to the Snellen chart for all cases that were controlled. 

## 5. Discussion

There is a serious risk of amblyopia if the upper lid obscures the visual axis in children with congenital ptosis [13]. Therefore, surgical operation should be performed immediately to decrease the risk of amblyopia [10, 13]. If levator function is less than 4 mm, the most common surgical approach is the frontal suspension technique [13]. There is a consensus regarding the use of this method [4, 9, 13], but different materials are used as suspension materials. The most successful material is autogenous fascia lata, and this technique has the lowest complication rate [3, 13]. Crawford, however, reported that it was difficult to harvest fascia lata in patients younger than 3 years old and that even if it could be harvested, the amount of material would not be sufficient for the operation [13]. Therefore, the use of different materials for frontal suspension had to be considered.

One of these materials was banked fascia lata [4]. Crawford reported that more inflammatory reactions were observed with the banked fascia lata compared to autogenous fascia lata due to the suture material [13]. Wagner reported that no infections or granulomas were observed with banked fascia lata, but the recurrence rate was 8.3% [14]. Wilson and Johnsonreported that the success rate decreased gradually and reached 50% by 9 years after the surgery [4]. Wasserman et al. reported that the recurrence rate was 4.2% with autogenous fascia lata and 51.4% with banked fascia lata [9]. These studies show that banked fascia lata is practical and easy to use, but in the long term, its efficacy decreases. 

Synthetic materials are also used in frontal suspension, but the success and the complication rates are different in different studies [9, 13–15]. Carter et al. performed frontal suspension with silicon bands in a total of 61 eyes (17 of them congenital) [7] and reported excellent and good results after a mean follow-up period of 22 months, with only 4 eyes requiring revision. Silicon band extrusion, however, was reported in 3 (5%) eyes. Polytetrafluoroethylene (Gore-Tex, AZ, USA) is a biologically and chemically inert substance used as a frontal suspension material. Steinkogler et al. used Gore-Tex in 37 eyes and reported only one recurrence [15], but the disadvantages of polytetrafluoroethylene were infection and granuloma formation [9, 14]. Wasserman et al. reported the rate of these complications to be 45.5% [9]. 4/0 nylon polyfilament suture (Supramid, Virginia, USA) is another synthetic material used as a suspension material before 3 years of age [8], but it has a high recurrence rate (29–69%) [8, 9, 14] due to degradation of thesupramidby hydrolysis after implantation [16]. Therefore, it is better to use this suture for temporary eyelid elevation [17]. Positive results have been obtained for all of these materials, but care should be taken due to the complication rates. 

Crawford reported that it was hard to harvest fascia lata in patients younger than 3 years old and that even if it could be harvested, the amount of material would not be sufficient for the operation [3]. Therefore, the use of different materials for frontal suspension had to be considered. Another reason to consider these materials is the possibility of functional and cosmetic leg problems due to fascia lata harvesting. There are not many studies regarding the use of autogenous fascia lata in this age group [10, 18]. Naugle et al. reported good results in two children using autogenous fascia lata, [18] and Leibovitch et al. performed frontal suspension with autogenous fascia lata in 14 eyes of 9 patients (6 months–2.5 years; mean age 15.3 months) with congenital ptosis [10]. Satisfying cosmetic and functional results were obtained in all patients with a mean follow-up period of 41.6 months [10]. No complications or recurrences were reported in any of the cases [10]. Only one hypertrophic scar case was reported in the leg of one patient [10]. 

In the present study, frontal suspension with autogenous fascia lata was performed on 43 eyes of 35 patients with congenital ptosis. To our knowledge, this is the largest series of patients in the literature. We did not experience any problems while harvesting the fascia lata and obtained enough fascia lata in all patients such that we did not need to use other materials due to insufficiency. Hematomas and muscle herniations can be complications due to the removal of fascia lata, but we did not experience these problems since we harvested the fascia lata using small incisions with a stripper. We believe that surgical experience is the most important factor to harvest a sufficient amount of fascia lata easily. Excellent anatomical knowledge and experience lead to successful results. We believe that consultations with orthopedic surgeons are useful for the first operation. 

There may be concerns about functional loss in the leg while harvesting fascia lata in children younger than 3 years old, but none of the parents reported these kinds of problems during the follow-up period. Leg pain and walking difficulties were reported in the first week postoperatively, but there were no complaints after the first week. The most important potential long-term problem is leg scars, but scars obtained early in life become less noticeable as the children grow, and no complaints were reported. The parents reported that it caused no problem after the autologous procedure was described to them. 

In summary, autogenous fascia lata can be used in all age groups. No other synthetic material is necessary when autogenous fascia lata is taken in sufficient amounts using the correct technique. We think that possible leg scars or the minimal complications in the early postoperative period are not important when we have a chance to use the autologous procedure.
